# Supplementary material for: The biological role of local and global fMRI BOLD signal variability in multiscale human brain organization
Source: Nat Commun. 2026 Jan 30;17:2189. doi: 10.1038/s41467-026-68700-0 (PMC12960827; doi:10.1038/s41467-026-68700-0)
Supplement: Supplementary file 1 — Supplementary Information [file 41467_2026_68700_MOESM1_ESM.pdf]

Supplemental Information

The biological role of local and global fMRI BOLD signal variability in multiscale human brain organization

Giulia Baracchini[1*], Yigu Zhou[1], Jason da Silva Castanheira[1], Justine Y. Hansen[1], Can Fenerci[2], Roni Setton[3], Jenny Rieck[4], Gary R. Turner[5], Cheryl L. Grady[6], Bratislav Misic[1], Jason Nomi[7], Lucina Q. Uddin[7], R. Nathan Spreng[1*]

Affiliations:
[1]Montreal Neurological Institute, Department of Neurology and Neurosurgery, McGill University, Montreal, QC, Canada
[2]Department of Psychology, McGill University, Montreal, QC, Canada
[3]Department of Psychology, Harvard University, Cambridge, MA, USA
[4]Health Canada, Ottawa, ON, Canada
[5]Department of Psychology, York University, Toronto, ON, Canada
[6]Rotman Research Institute at Baycrest, and Department of Psychiatry and Psychology, University of Toronto, Toronto, ON, Canada
[7]Department of Psychiatry and Biobehavioral Sciences, University of California Los Angeles, Los Angeles, USA

***Correspondence:**

Giulia Baracchini: giulia.baracchini@mail.mcgill.ca
R. Nathan Spreng: nathan.spreng@mcgill.ca

Consistency of covSTATIS results across multiple window lengths

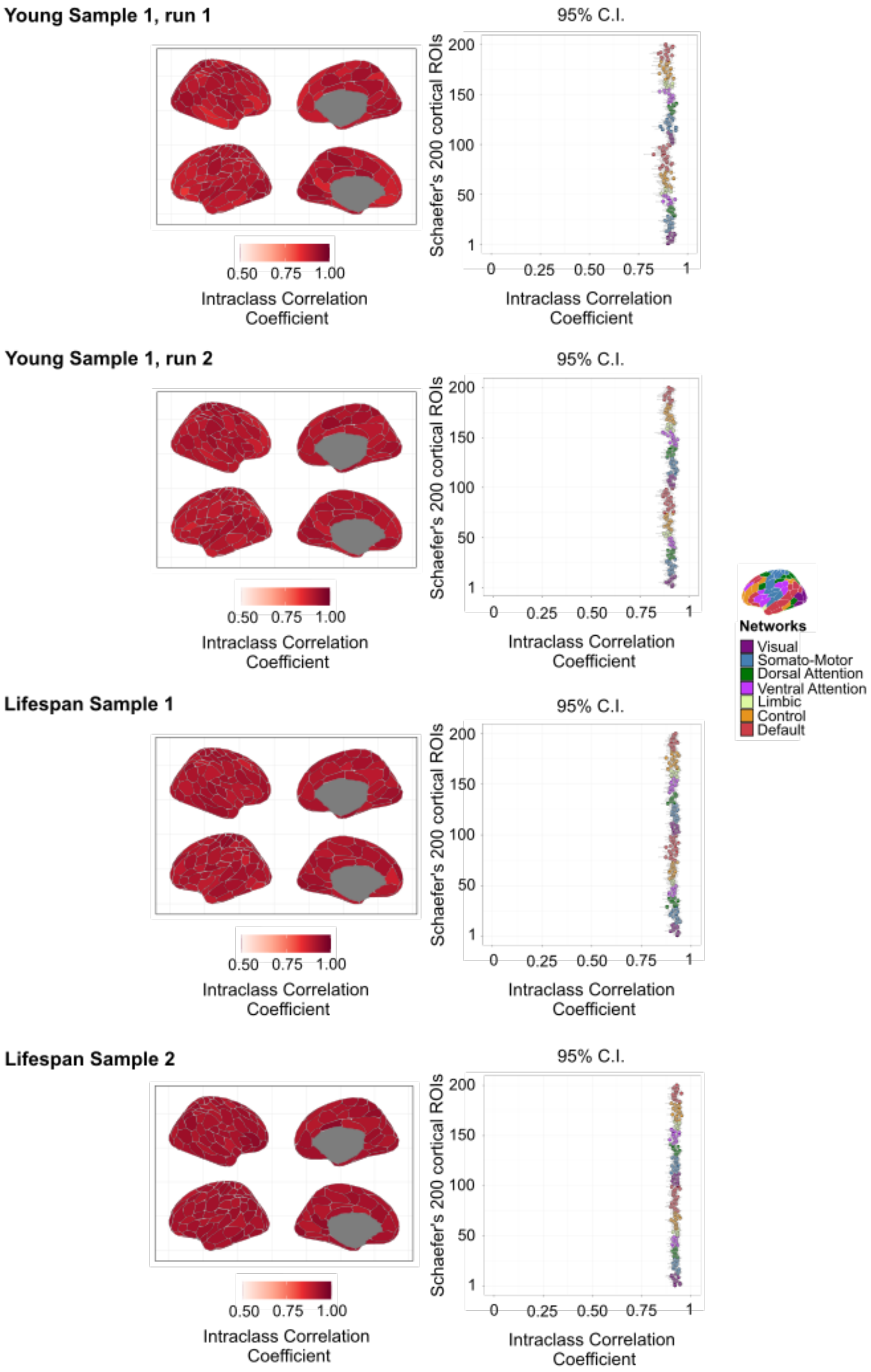

Figure S1. CovSTATIS shows consistent results across windows of different length. We calculated global BOLD signal variability (i.e., covSTATIS' area of the hull values) for each fMRI sample across windows of different length (45sec, 60sec, 80sec, 100sec; see Methods for full details). For each sample, we assessed inter-window consistency via intraclass coefficient statistics (ICC) and their 95% confidence intervals. Specifically, within each sample, ICCs were computed for each region (n=200) and were derived via a mean-rating (k=4 window lengths), consistency, two-way mixed effects model with window size as fixed effect and individuals as random term (Young Sample 1-run 1 n=150, Young Sample 1-run 2 n=145, Lifespan Sample 1 n=154, Lifespan Sample 2 n=154). ICC values were greater than 0.82 for all regions in all samples, indicating high consistency of covSTATIS estimates. Source Data, with exact ICC and C.I. values, are provided as a Source Data file.

Validation of covSTATIS age effects across multiple window lengths

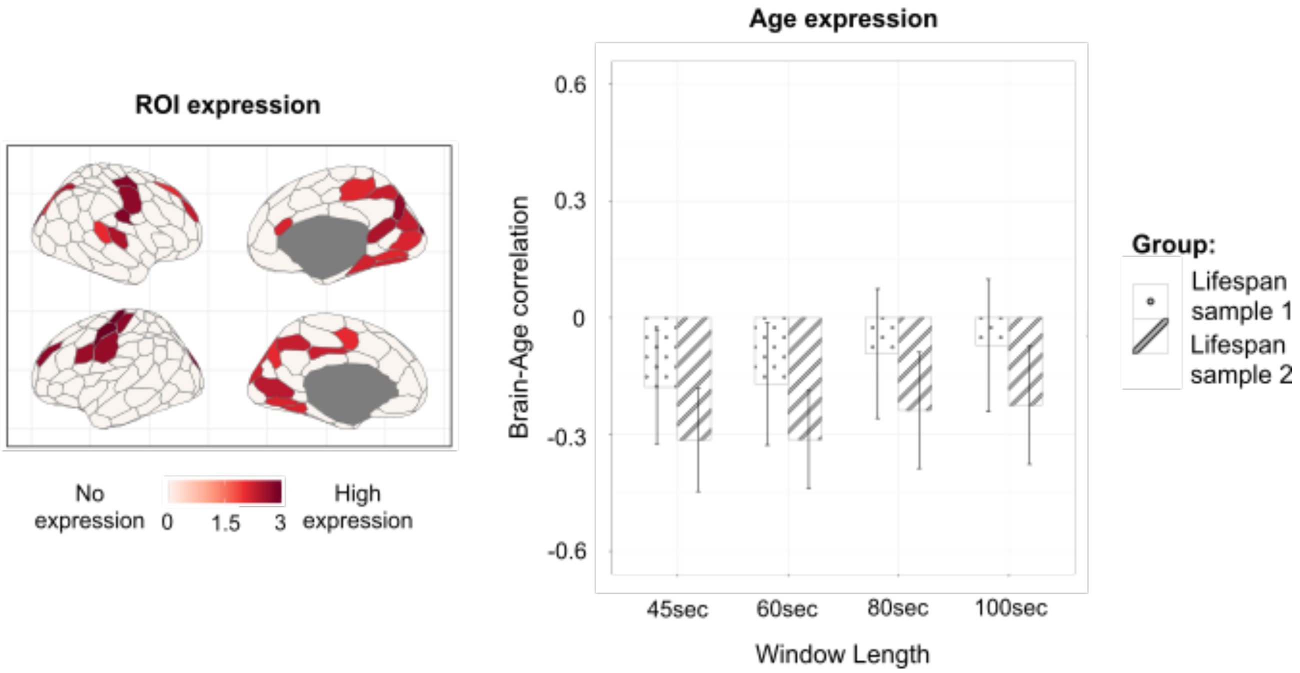

Figure S2. CovSTATIS-derived age effects are stable across windows of different length. To assess the stability of covSTATIS age effects, we ran our PLS analyses contrasting covSTATIS values for four window lengths across the two Lifespan Samples (i.e., a 2-group 4-condition design; see Methods for full details). We found age effects to be stable both in terms of their spatial location (brain plot on the left) and directionality (bar plot on the right; LV1 at $p<.001$ explaining 58% brain-age variance – significance obtained via permutation testing 1000x). Error bars indicate 95% confidence intervals from 1000 bootstrapped samples. Source Data are provided as a Source Data file.

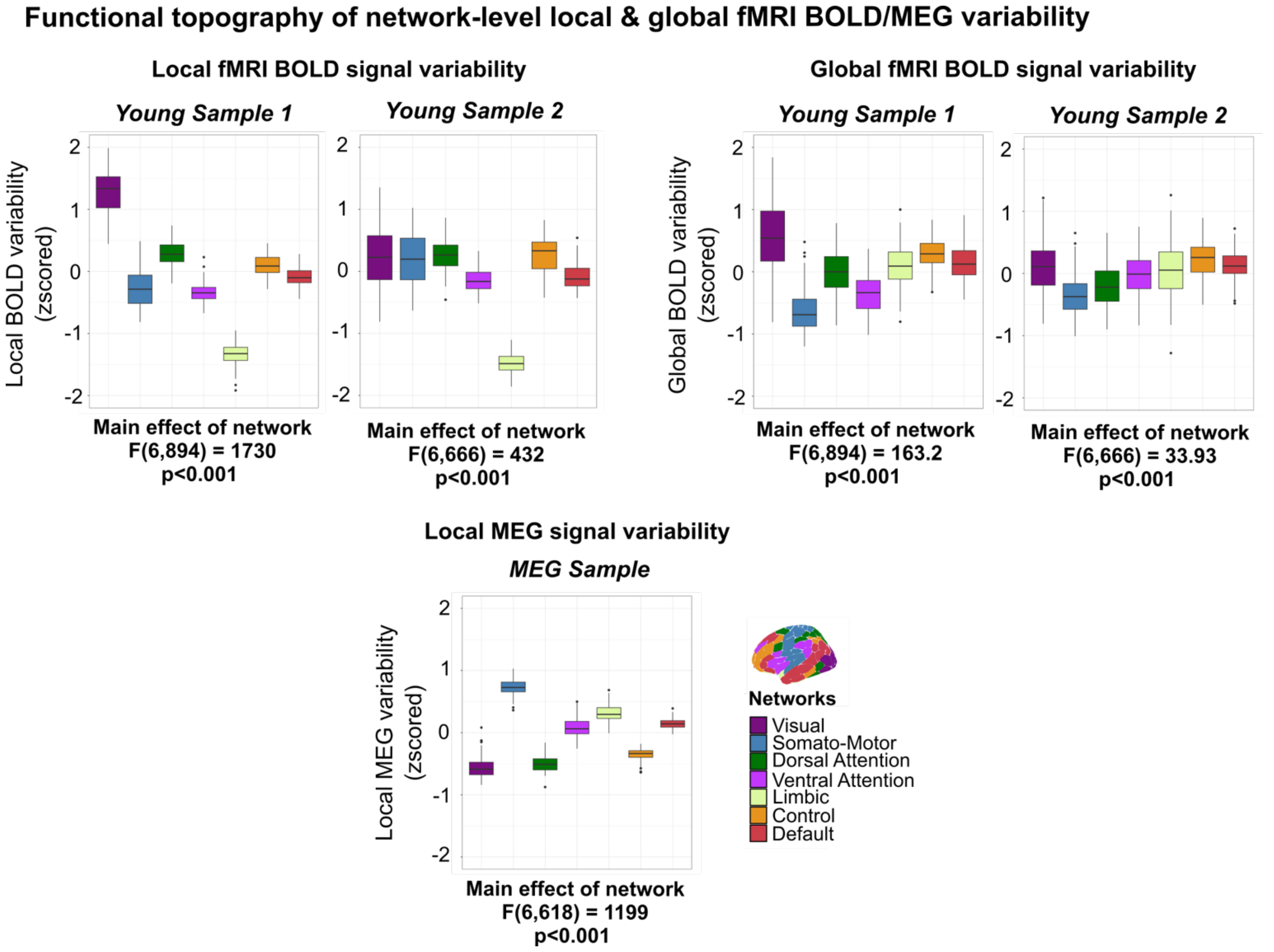

Figure S3. Topographical network-level characterization of local and global brain signal variability of fMRI and MEG data. Regional rMSSD and covSTATIS values were first scaled within each individual for each sample and then averaged within the 7 canonical functional brain networks. This yielded subject-level network means (e.g., Young Sample 1: n=150 subjects, 7 networks per subject). To quantify network differences, within each sample, we ran a one-way repeated measures ANOVA with 'network' as a within-subject factor and 'subject' as a random factor (see figure for statistics). Boxplots show the distribution of subject-level network means, with the central line representing the median, the box bounds denoting the 25th-75th percentiles, the whiskers extending 1.5x the interquartile range, and the points beyond the whiskers showing outliers. These results highlight how local BOLD signal variability exhibited greater topographical divergence between fMRI samples than global BOLD signal variability and showed the greatest variation in network topography by fMRI data type. Source Data are provided as a Source Data file.

Rank differences in the functional topography of local & global BOLD variability between fMRI data

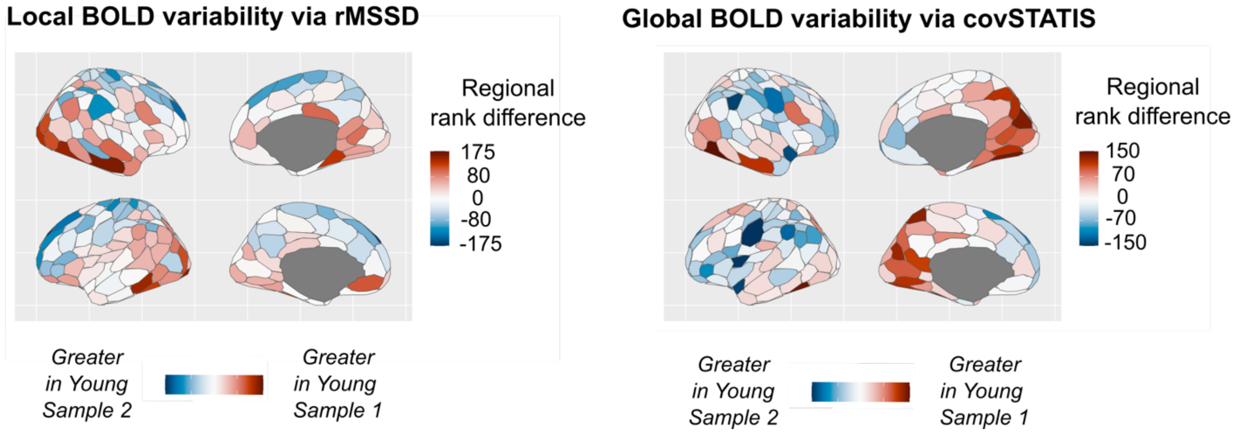

Figure S4. Regional rank differences in local and global BOLD signal variability across fMRI datasets. Regional values (n=200) of local and global BOLD variability were independently ordered within each fMRI dataset (Young Sample 1 and 2), and the difference in their ranks was derived for each of the 200 cortical regions. Positive rank values indicate greater local and global BOLD signal variability in Young Sample 1, while negative rank values indicate greater local and global BOLD signal variability in Young Sample 2. A value of zero indicates perfect correspondence in rank order between the two fMRI samples. These descriptive results show that higher-order cortices exhibit greater inter-sample correspondence for both local and global BOLD signal variability, than lower-order regions. Source Data are provided as a Source Data file.

Local BOLD variability relates to layer IV & global BOLD variability to layers V-VI thickness

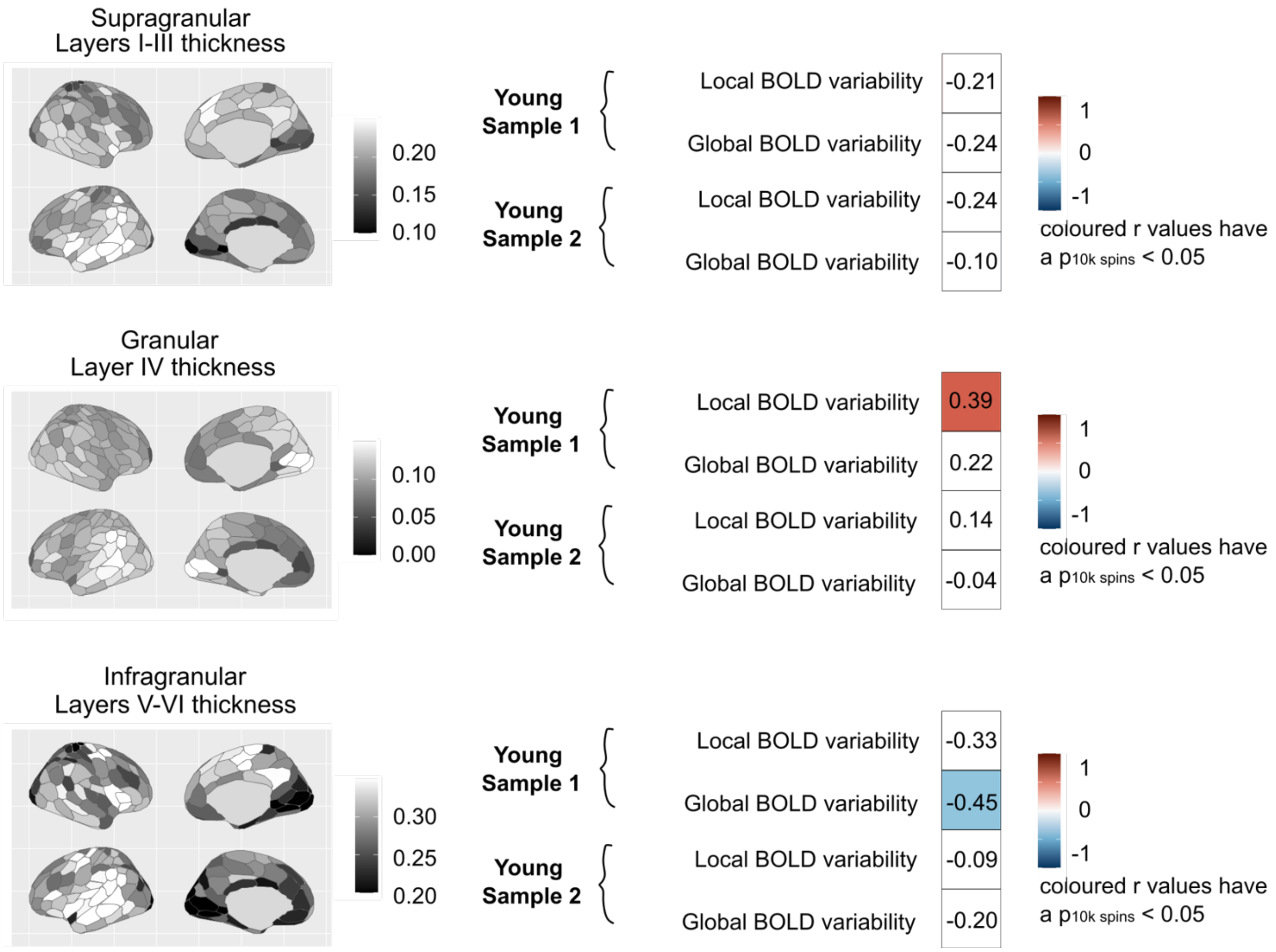

Figure S5. Relationships between cortical layer thickness (I-VI) and local and global BOLD signal variability for each fMRI dataset. We derived mean supragranular, granular and infragranular thickness maps from averaging thickness values in layers I-III, layer IV, layer V-VI retrieved from the BigBrainWarp toolbox (see Methods; left brain maps showing regional (n=200) mean thickness values for each set of layers, with the grey scale bar ranging from thinner (lighter) to thicker (darker) values). For each fMRI Young Sample, we then computed Pearson's product-to-moment correlations between these measures and group-level maps of local and global BOLD signal variability (one value per region). Significance was assessed via two-sided spin tests based on 10,000 Hungarian permutations of the regional labels of local and global BOLD variability. On the right, tables show resulting Pearson's correlation values split by metric and sample. Colored boxes indicate significant correlations ($p_{10k\ spin}<0.05$). Source Data are provided as a Source Data file.

A **BOLD variability association patterns integrating Margulies Gradient 2**

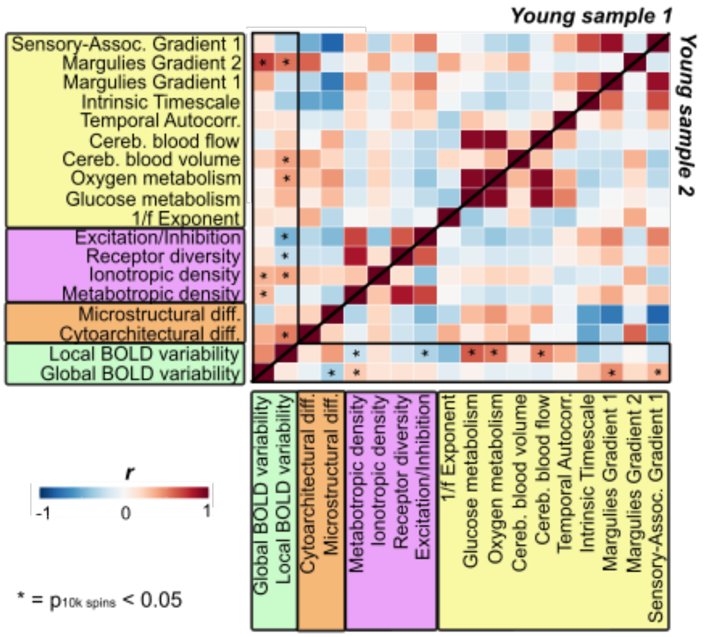

B **BOLD variability prediction patterns integrating Margulies Gradient 2**

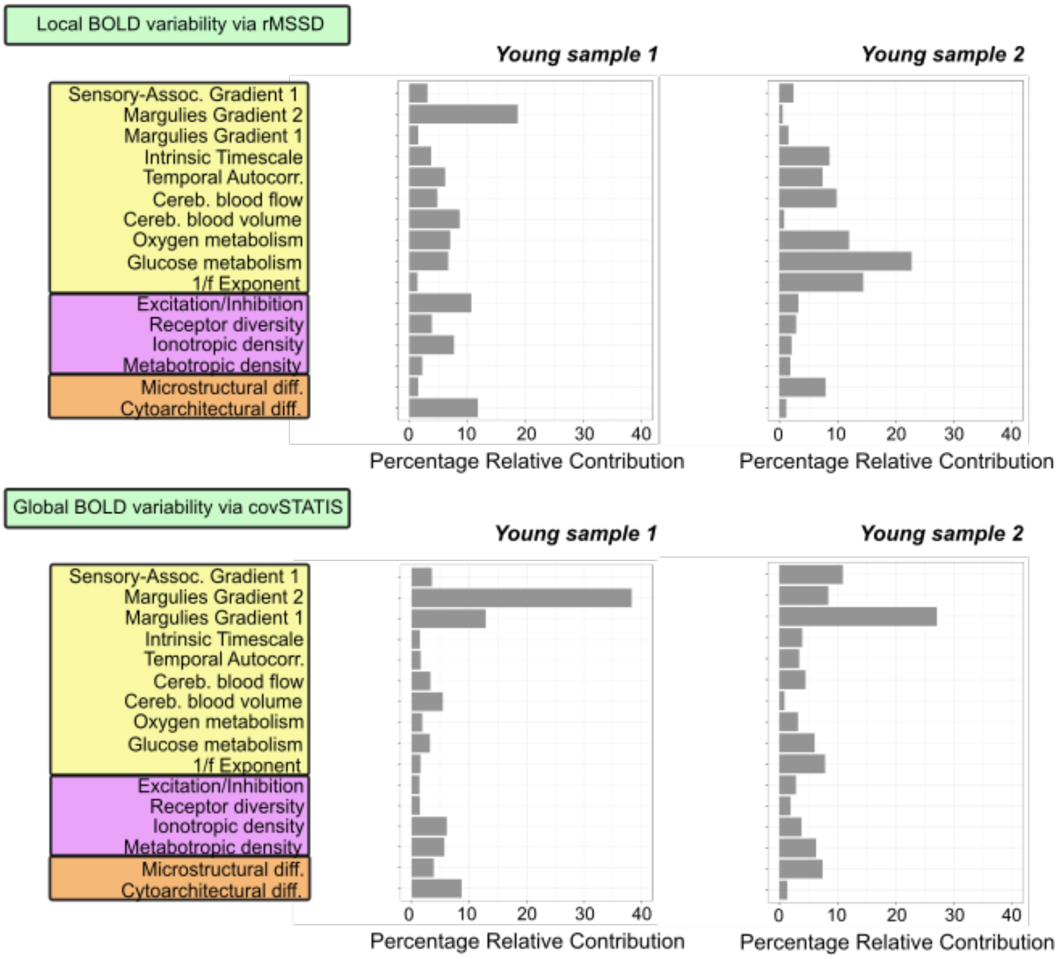

Figure S6. Multiscale neurobiological correlates of local and global BOLD signal variability including the second functional gradient. | (A) Correlation matrices for each fMRI sample (upper triangle: Young Sample 1; lower triangle: Young Sample 2) computed using Pearson's product-to-moment correlation. Asterisks indicate correlations that survived significance testing (10,000 Hungarian spins of Schaefer's regional labels; two-sided spin permutation test). We found positive associations between local BOLD signal variability and the second functional connectivity gradient (r=0.54, $p_{10k\ spin}$=0.03 Young Sample 1), and global BOLD signal variability and the second gradient (r=0.70, $p_{10k\ spin}$<0.001 Young Sample 1). As expected, participation coefficient scores of local and global BOLD variability including the second gradient largely overlapped with the values obtained by including only the first gradient (Young Sample 1: local = 0.62, global = 0.66; Young Sample 2: local = 0.49, global = 0.57). Additionally, in probing the inter-sample convergence of multiscale variability patterns, we found a significant inter-sample correlation only for local BOLD variability (Fisher-z transformed Pearson's r=0.64, p=0.007, 95% C.I. [0.22, 0.86]) and not for global BOLD variability (Fisher-z transformed Pearson's r=0.49, p=0.05, 95% C.I. [-0.001, 0.80]), differently from what we reported on the first gradient alone. **| (B)** Dominance analysis results (percent variance explained) per metric and fMRI sample including the second functional gradient. Source Data are provided as a Source Data file.

Hierarchical Linear Model: relationship between 1/f and local MEG variability

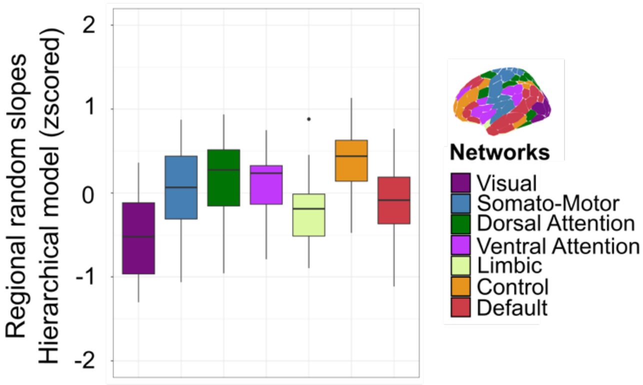

	Exponent		
Predictors	*Estimates*	*CI*	*p*
(Intercept)	1.10	1.08 – 1.11	**<0.001**
Local MEG Variability	-1.29	-1.41 – -1.17	**<0.001**
**Random Effects**			
σ^2	0.01		
τ_{00} Regions	0.01		
τ_{11} Regions.Local_MEG_Variability	0.42		
ρ_{01} Regions	-0.83		
ICC	0.32		
N Regions	200		
Observations	20800		
Marginal R^2 / Conditional R^2	0.106 / 0.388		

Figure S7. Topographical variation in the relationship between local MEG variability and the 1/f exponent. To test for topographical heterogeneity in the associations between local MEG variability and the 1/f exponent, we built a hierarchical linear model with regions as random effects. Specifically, we modelled the 1/f exponent as the dependent variable, local MEG variability as a fixed effect, and allowed both intercepts and slopes to vary across regions (104 subject x 200 cortical regions). All statistical tests were two-sided. Model statistics are presented in the table on the right. On the left, regional model slopes were averaged within the 7 canonical functional networks to show topographical variation in the association between local MEG variability and the 1/f exponent. Boxplots show the following information: the centre line represents the median, the box bounds indicate the 25th and 75th percentiles, the whiskers extend to 1.5x the interquartile range, and the points outside the whiskers represent outliers (each point is a region-specific slope value). Source Data are provided as a Source Data file.
