# Supplementary material for: The biological role of local and global fMRI BOLD signal variability in multiscale human brain organization
Source: Nat Commun. 2026 Jan 30;17:2189. doi: 10.1038/s41467-026-68700-0 (PMC12960827; doi:10.1038/s41467-026-68700-0)
Supplement: Supplementary file 2 — Reporting Summary [file 41467_2026_68700_MOESM2_ESM.pdf]

nature portfolio

Corresponding author(s): Giulia Baracchini, R. Nathan Spreng

Last updated by author(s): Dec 9, 2025

Reporting Summary

Nature Portfolio wishes to improve the reproducibility of the work that we publish. This form provides structure for consistency and transparency in reporting. For further information on Nature Portfolio policies, see our Editorial Policies and the Editorial Policy Checklist.

Statistics

For all statistical analyses, confirm that the following items are present in the figure legend, table legend, main text, or Methods section.

n/a	Confirmed	
☐	☒	The exact sample size (*n*) for each experimental group/condition, given as a discrete number and unit of measurement
☐	☒	A statement on whether measurements were taken from distinct samples or whether the same sample was measured repeatedly
☐	☒	The statistical test(s) used AND whether they are one- or two-sided<br>*Only common tests should be described solely by name; describe more complex techniques in the Methods section.*
☒	☐	A description of all covariates tested
☐	☒	A description of any assumptions or corrections, such as tests of normality and adjustment for multiple comparisons
☐	☒	A full description of the statistical parameters including central tendency (e.g. means) or other basic estimates (e.g. regression coefficient) AND variation (e.g. standard deviation) or associated estimates of uncertainty (e.g. confidence intervals)
☐	☒	For null hypothesis testing, the test statistic (e.g. *F*, *t*, *r*) with confidence intervals, effect sizes, degrees of freedom and *P* value noted<br>*Give P values as exact values whenever suitable.*
☒	☐	For Bayesian analysis, information on the choice of priors and Markov chain Monte Carlo settings
☒	☐	For hierarchical and complex designs, identification of the appropriate level for tests and full reporting of outcomes
☐	☒	Estimates of effect sizes (e.g. Cohen's *d*, Pearson's *r*), indicating how they were calculated

Our web collection on statistics for biologists contains articles on many of the points above.

Software and code

Policy information about availability of computer code

Data collection	No data were collected by the authors for this study (and hence no software was used for data collection).
Data analysis	This manuscript relied on preprocessed fMRI and MEG data and used various open access resources. fMRI data from Young Sample 1 were preprocessed using ME-ICA v3.2 (https://github.com/ME-ICA/me-ica) via AFNI. fMRI data from Young Sample 2 and Lifespan Sample 2 were preprocessed using the DPARSF-A toolbox v4.3_170105 (http://rfmri.org/DPARSF). fMRI data from Lifespan Sample 1 were preprocessed using the Optimising of Preprocessing Pipelines for NeuroImaging Software Package (OPPNI, available at https://github.com/strotherlab/oppni). Movie watching fMRI data from the fMRI-MEG dataset were preprocessed using fMRIprep 21.0.1 and tedana v2.5. MEG data were preprocessed using Brainstorm (March 2021 distribution). All data analyses and the data presented in the figures, except for the electrophysiological timeseries simulations, were obtained in R v4.2-4.3 and MATLAB R2022b, R2024b. Partial Least Squares analyses were carried out using an openly available MATLAB toolbox (https://github.com/McIntosh-Lab/PLS/). Simulated naturalistic electrophysiological timeseries were generated using the NeuroDSP toolbox (https://neurodsp-tools.github.io/neurodsp). Custom code is available at https://github.com/giuliabaracc/BiologicalVariability.

For manuscripts utilizing custom algorithms or software that are central to the research but not yet described in published literature, software must be made available to editors and reviewers. We strongly encourage code deposition in a community repository (e.g. GitHub). See the Nature Portfolio guidelines for submitting code & software for further information.

Data

Policy information about availability of data

All manuscripts must include a data availability statement. This statement should provide the following information, where applicable:

- Accession codes, unique identifiers, or web links for publicly available datasets
- A description of any restrictions on data availability
- For clinical datasets or third party data, please ensure that the statement adheres to our policy

Resting-state fMRI data from Young Sample 1 can be accessed on OpenNeuro at the following link: https://openneuro.org/datasets/ds003592/versions/1.0.13. Resting-state fMRI data from Young Sample 2 and Lifespan Sample 2 are available for download at the following link: http://fcon_1000.projects.nitrc.org/indi/enhanced/. fMRI-MEG data can be accessed by requesting the data at: https://www.cam-can.org/index.php?content=dataset. Microscale neurobiological data are downloadable from the BigBrainWarp Toolbox: https://bigbrainwarp.readthedocs.io/en/latest/pages/installation.html. Mesoscale and macroscale neurobiological data are retrievable from the Abagen and Neuromaps toolboxes: https://github.com/netneurolab/abagen; https://github.com/netneurolab/neuromaps.

Research involving human participants, their data, or biological material

Policy information about studies with human participants or human data. See also policy information about sex, gender (identity/presentation), and sexual orientation and race, ethnicity and racism.

Reporting on sex and gender	While gender information was not available to us, we reported in text, for all human neuroimaging samples included, sex information as follows: 55% of Young Sample 1, 54% of Young sample 2, 62% of Lifespan Sample 1 and 2, and 56% of our fMRI-MEG sample were female. We balanced sex across samples matched by age. All analyses were run on both sexes together.
Reporting on race, ethnicity, or other socially relevant groupings	Information regarding race, ethnicity or other socially relevant groupings was not available to us, hence why we did not run analyses testing for specific race/ethnicity effects.
Population characteristics	All human neuroimaging data included in this study were collected on healthy adults. We selected participants ages 18-34 from fMRI Young Samples 1 & 2 and the fMRI-MEG sample, and ages 20-86 from fMRI Lifespan Samples 1 & 2. Histological data were collected post-mortem from a male of 65 years of age. Microstructural data were derived from a healthy adult sample of 50 individuals ages 23-35. Population characteristics for neurotransmitter receptor and metabolic data can be found in Table 1 of Hansen et al., 2022 Nature Neuroscience.
Recruitment	Only data from healthy adults were used in the study.
Ethics oversight	All research protocols from data used in this study were approved by Ethics Institutional Review Boards, as follows: Cornell University Board for Young Sample 1, NKI board for Young Sample 2 and Lifespan Sample 2, Ethics board at Baycrest Health Sciences Center for Lifespan Sample 1, Cambridgeshire 2 Ethics Committee for fMRI-MEG data, and the Ethics Committee of the Montreal Neurological Institute and Hospital for microstructural data.

Note that full information on the approval of the study protocol must also be provided in the manuscript.

Field-specific reporting

Please select the one below that is the best fit for your research. If you are not sure, read the appropriate sections before making your selection.

☒ Life sciences ☐ Behavioural & social sciences ☐ Ecological, evolutionary & environmental sciences

For a reference copy of the document with all sections, see nature.com/documents/nr-reporting-summary-flat.pdf

Life sciences study design

All studies must disclose on these points even when the disclosure is negative.

Sample size	Sample sizes were determined based on the availability of healthy participants' data in each openly accessible dataset. No statistical methods were used to predetermine sample sizes. Instead, we included all eligible individuals that met the age-range criteria established from our primary datasets (Young Sample 1: 18-34 years; Lifespan Sample 1: 20-86 years). As such, Young Sample 2 and the fMRI-MEG sample were age-matched to Young Sample 1, and Lifespan Sample 2 was age-matched to Lifespan Sample 1. For Young Sample 1, all available participants were included (n = 150 for run 1; of which n = 145 had a second run). Young Sample 2 comprised of n = 112 age-matched individuals. Lifespan Sample 1 included n = 154 individuals across the adult lifespan, and Lifespan Sample 2 was matched to this distribution (n = 154). For the fMRI-MEG dataset, all individuals with available data were included (MEG: n = 104; of which n = 103 also had fMRI). Neurobiological datasets were also used in full, specifically: histology (n = 1), microstructure (n = 50 individuals), and neurotransmitter and metabolic maps as provided in Table 1 of Hansen et al., 2022 (Nature Neuroscience).These sample sizes are sufficient for the goals of the study because the main analyses rely on group-level spatial correlation, and are replicated across independent samples.
Data exclusions	For Young Sample 1, we included all data from young adults ages 18-34. For Young Sample 2, we only included data from young adults matched by age to Young Sample 1. For Lifespan Sample 1, we included all data from adults ages 20-86. For Lifespan Sample 2, we only included data from individuals that matched by age Young Sample 2. For our MEG sample, we only included data from individuals in the 18-34 age range. For the fMRI portion of the fMRI-MEG sample, we included 103 individuals to test for fMRI-MEG relationships within sample. For all other neurobiological data, we used what was available online.

Replication	We specifically decided to run our fMRI analyses on 4 datasets (Young Samples 1 & 2, Lifespan Samples 1 & 2) to test for generalizability of findings. Results generalized across samples (except for difference in reliability between Young Sample 1 & 2 due to different fMRI data type). Additionally, we replicated cross-sample fMRI-MEG comparisons using within-individual fMRI-MEG data.
Randomization	Not relevant to this study, since we did not include experimental groups.
Blinding	Not relevant to this study, since we did not include experimental groups.

Reporting for specific materials, systems and methods

We require information from authors about some types of materials, experimental systems and methods used in many studies. Here, indicate whether each material, system or method listed is relevant to your study. If you are not sure if a list item applies to your research, read the appropriate section before selecting a response.

Materials & experimental systems

n/a	Involved in the study
☒	☐ Antibodies
☒	☐ Eukaryotic cell lines
☒	☐ Palaeontology and archaeology
☒	☐ Animals and other organisms
☒	☐ Clinical data
☒	☐ Dual use research of concern
☒	☐ Plants

Methods

n/a	Involved in the study
☒	☐ ChIP-seq
☒	☐ Flow cytometry
☐	☒ MRI-based neuroimaging

Plants

Seed stocks	*Report on the source of all seed stocks or other plant material used. If applicable, state the seed stock centre and catalogue number. If plant specimens were collected from the field, describe the collection location, date and sampling procedures.*
Novel plant genotypes	*Describe the methods by which all novel plant genotypes were produced. This includes those generated by transgenic approaches, gene editing, chemical/radiation-based mutagenesis and hybridization. For transgenic lines, describe the transformation method, the number of independent lines analyzed and the generation upon which experiments were performed. For gene-edited lines, describe the editor used, the endogenous sequence targeted for editing, the targeting guide RNA sequence (if applicable) and how the editor was applied.*
Authentication	*Describe any authentication procedures for each seed stock used or novel genotype generated. Describe any experiments used to assess the effect of a mutation and, where applicable, how potential secondary effects (e.g. second site T-DNA insertions, mosiacism, off-target gene editing) were examined.*

Magnetic resonance imaging

Experimental design

Design type	Resting-state fMRI (except for the fMRI-MEG sample where fMRI data were collected during movie watching)
Design specifications	All fMRI datasets included one run of fMRI, except Young Sample 1 from which we included two runs.
Behavioral performance measures	No behavioural performance measures were included, since all data used are resting-state or passive movie watching.

Acquisition

Imaging type(s)	Functional
Field strength	3 Tesla
Sequence & imaging parameters	Young Sample 1: multi-echo EPI sequence with online reconstruction (TR=3000 ms; TE1=13.7 ms, TE2=30 ms, TE3=47 ms; 83° flip angle; matrix size=72 × 72; FOV=210 mm; 46 axial slices; 3mm isotropic voxels; 204 volumes) with 2.5× acceleration and sensitivity encoding.<br>Young Sample 2 & Lifespan Sample 2: multiband (factor of 4) EPI sequence (TR=1400 ms; TE=30 ms; 65° flip angle; FOV=224mm; 64 axial slices; 2mm isotropic voxels; 404 volumes).<br>Lifespan Sample 1: EPI sequence (TR=2000 ms; TE=27 ms; 70° flip angle; FOV=192mm; 40 axial slices; 3mm isotropic voxels; 297 volumes).<br>fMRI from fMRI-MEG sample: multi-echo T2* EPI sequence with 193 total volumes, 32 axial slices, 3.7 mm thick, 0.74 mm gap, TR = 2470 msec, TE = [9.4, 21.2, 33, 45, 57] msec, flip angle = 78°, FOV =192x192mm, voxel size = 3x3x4.44mm.
Area of acquisition	Whole-brain

Diffusion MRI ☐ Used ☒ Not used

Preprocessing

Preprocessing software	fMRI data from Young Sample 1 were preprocessed using ME-ICA v3.2 (https://github.com/ME-ICA/me-ica): skull stripping in BET, minimal preprocessing (the first 4 volumes were discarded, images were computed for de-obliquing, motion correction, and anatomical-functional coregistration, and volumes were brought into spatial alignment across TEs), denoising via PCA and ICA. fMRI data from Young Sample 2 and Lifespan Sample 2 were preprocessed using DPARSF-A (Yan and Zang, 2016 Neuroinformatics): removal of the first 5 volumes, despiking using AFNI 3dDespike, realignment, normalization to 3 mm MNI template, and smoothing (6 mm FWHM), denoising via ICA-FIX, nuisance covariance regression (linear detrend, Friston 24 motion parameters (6 motion parameters of each volume, the preceding volume, and the 12 corresponding squared items), followed by bandpass filtering (0.01–0.10 Hz). fMRI data from Lifespan Sample 1 were preprocessed using a combination of FSL tools: brain masks were created using BET, functional volumes across the time-series were co-registered to correct for motion (using FEAT in FSL), temporal detrending and bandpass filtering (0.01–0.10 Hz), MELODIC (in FSL) was used to compute independent components and dimensionality was estimated with the Laplace method so that ICA-based denoising with FIX could be conducted. Additional regression of motion parameters and signal in tissue of no interest (white matter, vessels, and cerebrospinal fluid) was also conducted. Finally, the data were smoothed with a 7mm^3 kernel, warped to MNI space, and resampled to 4mm^3 isotropic voxels.<br>fMRI data from the fMRI-MEG sample were minimally preprocessed using fMRIPrep. Head-motion parameters with respect to the BOLD reference (i.e., transformation matrices, and six corresponding rotation and translation parameters) were estimated. BOLD runs were slice-time corrected to 1.2 s (0.5 of slice acquisition range 0–2.4 s) using 3dTshift from AFNI. The BOLD time-series (including slice-timing correction when applied) were resampled onto their original, native space by applying the transforms to correct for head-motion. The minimally pre-processed outputs of fMRIPrep were then submitted to tedana. Multi-echo data were optimally combined, after which principal component analysis and the 'stabilized' Kundu component selection decision tree was applied for dimensionality reduction. Independent component analysis was then used to decompose the dimensionally reduced dataset. Additional steps were taken to reduce the effects of motion on the functional connectivity results. Wavelet despiking was applied to remove residual motion artefacts following tedana. Denoised and wavelet despiked data were lastly quality checked.
Normalization	fMRI data from Young Sample 1 were first linearly registered to each individual's T1-weighted image, and then the inverse of this registration was used to project the T1-weighted image to native space and resample the data onto a cortical surface (fsaverage5) with trilinear volume-to-surface interpolation. fMRI data from all other samples was normalized to standard MNI space.
Normalization template	MNI152
Noise and artifact removal	See above in Preprocessing software.
Volume censoring	Since we were interested in temporal variability measures, no volume censoring was applied.

Statistical modeling & inference

Model type and settings	Local fMRI BOLD variability: univariate via root Mean Square of Successive Differences metric (rMSSD, first-order temporal derivative). Global fMRI BOLD variability: multivariate via covSTATIS (sliding window of connectivity, Rv similarity coefficient, eigenvalue decomposition, back-projection of factor scores, Euclidean distance, area of hull). Validation of covSTATIS: multivariate via Partial Least Squares (PLS; covariance, singular value decomposition, permutations/bootstraps). Reliability of local & global BOLD variability: univariate via t-tests. Multiscale mapping of local/global BOLD variability: univariate via Pearson's correlation + spin test, participation coefficient, predictive modeling via dominance analysis. MEG & multimodal analyses: univariate.
Effect(s) tested	Temporal variability of resting-state fMRI via rMSSD and covSTATIS. Validation of covSTATIS as a method to assess global fMRI BOLD variability via PLS. Inter-sample regional and network stability of variability via t-tests. Integral role of variability via multiscale and multimodal analyses reported above.

Specify type of analysis: ☐ Whole brain ☐ ROI-based ☒ Both

Anatomical location(s): Schaefer 200-17 functional parcellation + Group Prior Individual Parcellation for Young Sample 1.

Statistic type for inference (See Eklund et al. 2016)	whole-brain and region-wise.
Correction	not applicable here.

Models & analysis

n/a	Involved in the study
☐	☒ Functional and/or effective connectivity
☐	☒ Graph analysis
☐	☒ Multivariate modeling or predictive analysis

Functional and/or effective connectivity	Pairwise Pearson correlation between regions.
Graph analysis	Participation coefficient on multiscale correlation matrices: we first assigned (1) local and global BOLD variability, (2) microscale variables, (3) mesoscale and (4) macroscale measures to four different communities. We then took the absolute value of the reported correlations and calculated, for each fMRI sample, the participation coefficient of local and global BOLD variability.
Multivariate modeling and predictive analysis	(1) To obtain global measures of BOLD signal variability, we applied covSTATIS on sliding window connectivity matrices. As an extension of Principal Component Analysis, covSTATIS is a multidimensional scaling method that uses eigenvalue decomposition and Euclidean distance to evaluate the similarity of multiple data tables derived from the same set of observations (here connectivity matrices over time).<br>(2) We ran a Partial Least Squares (PLS) analysis between covSTATIS-derived areas of the hull (independent variables) and age (dependent variable). PLS calculates a covariance matrix between two (or more) sets of variables. This covariance matrix undergoes singular value decomposition and, as a result, orthogonal latent variables are generated (LVs; similar to principal components in PCA) which explain the covariance between the sets of measures. 1000 permutations and bootstraps were computed to assess significance of LVs and stability (95% CI) of results.<br>(3) We ran a dominance analysis to build a predictive model for each fMRI dataset, where we estimated the unique contribution of each neurobiological measure in predicting local and global BOLD variability. Dominance analysis allowed us to estimate the relative importance of each predictor in a single multiple regression model.
